# A Genomic Survey of Reb Homologs Suggests Widespread Occurrence of R-Bodies in Proteobacteria

**DOI:** 10.1534/g3.112.005231

**Published:** 2013-03-01

**Authors:** Kasie Raymann, Louis-Marie Bobay, Thomas G. Doak, Michael Lynch, Simonetta Gribaldo

**Affiliations:** * Institut Pasteur, Unité Biologie Moleculare du Gene chez les Extremophiles, Departement de Microbiologie, Paris, 75724 Cedex 15, France; † Université Pierre et Marie Curie, Cellule Pasteur UPMC, Paris, 75724 Cedex 15, France; ‡ Institut Pasteur, Microbial Evolutionary Genomics, Departement Genomes et Genetique, Paris, 75724 Cedex 15, France; § Centre National de la Recherche Scientifique, Unité Mixte de Recherche 3525, Paris, F-75015 France; ** Indiana University, Department of Biology, Bloomington, Indiana 47405

**Keywords:** kappa particles, *Caedibacter*, phylogenomics

## Abstract

Bacteria and eukaryotes are involved in many types of interaction in nature, with important ecological consequences. However, the diversity, occurrence, and mechanisms of these interactions often are not fully known. The obligate bacterial endosymbionts of Paramecium provide their hosts with the ability to kill sensitive Paramecium strains through the production of R-bodies, highly insoluble coiled protein ribbons. R-bodies have been observed in a number of free-living bacteria, where their function is unknown. We have performed an exhaustive survey of genes coding for homologs of Reb proteins (R-body components) in complete bacterial genomes. We found that *reb* genes are much more widespread than previously thought, being present in representatives of major Proteobacterial subdivisions, including many free-living taxa, as well as taxa known to be involved in various kinds of interactions with eukaryotes, from mutualistic associations to pathogenicity. Reb proteins display very good conservation at the sequence level, suggesting that they may produce functional R-bodies. Phylogenomic analysis indicates that *reb* genes underwent a complex evolutionary history and allowed the identification of candidates potentially involved in R-body assembly, functioning, regulation, or toxicity. Our results strongly suggest that the ability to produce R-bodies is likely widespread in Proteobacteria. The potential involvement of R-bodies in as yet unexplored interactions with eukaryotes and the consequent ecological implications are discussed.

During more than two billion years of coexistence, prokaryotes have established various forms of interaction with eukaryotes. Examples include the mutualistic symbioses that benefit eukaryotic host by providing nutrients, defense, competition, and adaptation to new environments (Gast *et al.* 2009). At the same time, bacteria have developed various ways to defend themselves against grazing by eukaryotes (Matz and Kjelleberg 2005), with potential implications for the emergence of pathogens (Brown and Barker 1999). However, the extent and diversity of bacterial/eukaryotic interactions in nature remains largely underexplored. As a growing amount of genomic data covering a large fraction of bacterial diversity becomes available, hints of such relationships may be gathered from *in silico* analyses. These can be linked to experimental observations, providing useful directions for further work.

A fascinating example of a bacterial/eukaryote relationship is provided by the killer endosymbionts of the ciliate Paramecium. In the 1930s Tracey Sonneborn discovered that some strains of the *Paramecium aurelia* complex have a killer phenotype toward sensitive strains (Beale and Preer 2004; Preer 2006; Sonneborn 1938). Sonneborn could show that this phenomenon is not controlled by nuclear genes, providing one of the first examples of cytoplasmic inheritance (Sonneborn 1943). It was later discovered that the killer phenotype is conveyed by an obligate endosymbiotic bacterium (also referred to as a kappa particle), and each killer paramecium strain harbors its own specific endosymbiont that usually resides in the cytoplasm but can also be in the nucleus (Preer 1975). Whereas all these obligate endosymbionts initially were placed in the genus Caedibacter, they were later shown to belong to different proteobacterial lineages (Beier *et al.* 2002).

In Caedibacter, the killer trait is directly linked to the production of R-bodies, unusual cytoplasmic refractile inclusion bodies (Dilts and Quackenbush 1986) [for review, see (Pond *et al.* 1989)]. What is known about R-bodies comes primarily from *Caedibacter taeniospiralis*, which belongs to the gammaproteobacteria family of Thiotrichales (Figure 1) (Beier *et al.* 2002). R-bodies are highly insoluble protein ribbons that are typically coiled into cylindrical structures. They are produced by only a fraction of the endosymbiont population, which then stop dividing. When R-body−containing bacteria are released into the environment and captured by sensitive strains, killing occurs very rapidly. The exact mechanism for killing is not known, but it is thought that internalization of the R-body-containing bacteria into the food vacuole triggers unrolling of the R-body, which penetrates the phagosomal membrane and delivers a killer toxin to the cytoplasm (Figure 1) (Jurand *et al.* 1971; Preer *et al.* 1974). Isolated R-body−containing bacteria are capable of killing sensitive Paramecium strains in various ways, with prelethal symptoms such as paralysis, vacuolization, and opposite swimming rotation. On the contrary, exposure to non-R-body−containing Caedibacter is not lethal [for review, see (Pond *et al.* 1989)], and a mutant Caedibacter strain unable to make R-bodies loses its killing ability (Dilts and Quackenbush 1986). Different strains of Caedibacter produce different types of R-bodies, which vary in diameter (0.25−0.8 mm), length (<10−30 mm), ribbon morphology (tapered or blunt termini), mode of unrolling (from the outside or from the inside in a telescopic fashion), and the nature of the stimulus for unrolling (changes in pH, temperature, ionic strength) [for review, see (Pond *et al.* 1989; Sanchez-Amat 2006)]. Interestingly, the unrolling of type 51 R-bodies has been shown to be reversible (they unroll when the pH is dropped <6.5 and reroll when the pH is again raised >7.0) (Pond *et al.* 1989).

**Figure 1:**
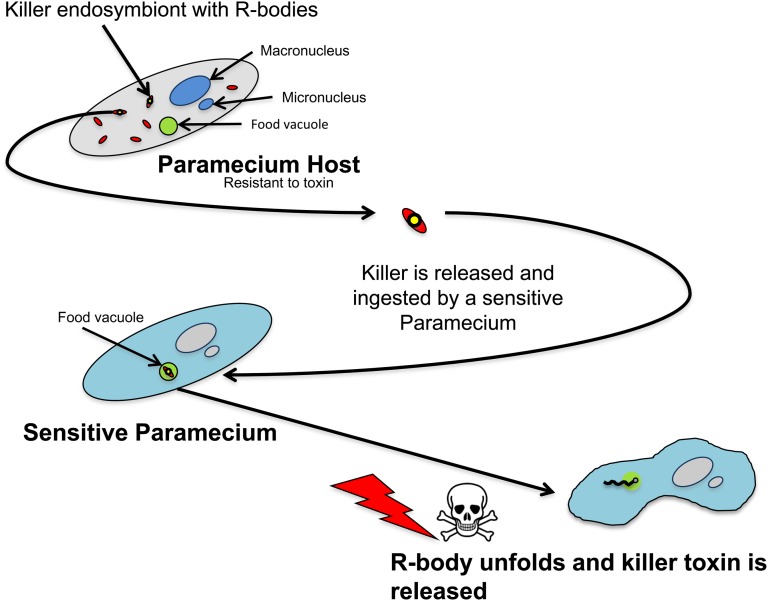
Illustration of the *C. taeniospiralis* R-body toxin delivery system (see main text for details and references).

Studies of the genetic determinants of R-bodies began in the early 1980s, and in *C. taeniospiralis* the R body−coding region was found to lie on a plasmid (Quackenbush and Burbach 1983). When a region from the pKAP47 plasmid (from *Paramecium teraurelia* California strain 47) was cloned into *Escherichia coli*, R-bodies were produced, but the clones did not exhibit toxicity toward sensitive Paramecium strains (Quackenbush and Burbach 1983). Therefore, whereas production of R-bodies is necessary for killing by *C. taeniospiralis*, as described previously, it is not sufficient for killing by recombinant *Escherichia coli*. This excludes a direct cytotoxic effect of R-bodies and indicates a requirement for an essential unknown toxin encoded either by the plasmid or the *C. taeniospiralis* genome (Preer and Stark 1953; Quackenbush and Burbach 1983). These data have been recently confirmed, and it has been shown that recombinant *E. coli* expressing the four *reb* genes of the *C. taeniospiralis* pKAP298 plasmid (from *Paramecium teraurelia* Panama strain 298) were capable of producing R-bodies but were not toxic toward sensitive Paramecium strains (Schrallhammer *et al.* 2012).

Very little information is available on the assembly process of R-bodies. At least three polypeptides of 10, 13, and 18 kDa were found to be involved in the structure and assembly of type 51 R-bodies (Kanabrocki *et al.* 1986) and were later shown to be encoded by three genes: *reb*C, *reb*B, and *reb*A, respectively, the last two being homologous (Heruth *et al.* 1994). These early data proposed that the major structural protein is RebB and that RebA may act as a scaffold to facilitate the polymerization process whereas RebC may act as a transcriptional regulator (Heruth *et al.* 1994). Finally, it was suggested that RebB might be modified posttranslationally, with the possible involvement of RebC (Heruth *et al.* 1994). The role of a fourth gene in the *reb* locus, *reb*D, coding for a homolog of RebA and RebB, is unclear but it was shown not to be necessary for R-body production in *E. coli* (Heruth *et al.* 1994).

The complete sequence of the Reb-harboring pKAP298 plasmid of *C. taeniospiralis* strain 298 was obtained in 2005 (Jeblick and Kusch 2005). It was found that this plasmid contains 63 open reading frames, 23 only having similarity with proteins with known function, and a few being similar to proteins encoded by phages or prophages, which led to the suggestion that the plasmid originated from a bacteriophage (Jeblick and Kusch 2005), which is consistent with early observations of the association of phage-like particles with R-bodies (Preer *et al.* 1974). A protein with homology to the Soj-ParA family of membrane-associated ATPases was suggested as a possible candidate for the toxin, which would kill the host by somehow affecting its membrane, although a precise mechanism was not proposed (Jeblick and Kusch 2005).

The harboring of an endosymbiont that produces R-bodies gives a competitive advantage to its killer Paramecium host with respect to sensitive strains (Kusch *et al.* 2002). In turn, R-body production seems to play a role in defense against predation and creates a benefit for the Caedibacter strains at the population level (Sanchez-Amat 2006). However, many important questions remain to be clarified. For example, it is not known how obligate symbiosis is established in the first place or how sensitive strains can pick up Caedibacter and become killers, nor how killer Paramecium strains are protected from their own specific R-body producing endosymbionts (Gibson 1973; Preer *et al.* 1974).

Interestingly, casual observations of coiled R-body structures of various types have been reported in several free-living bacteria: the hydrogen-oxidizing β-proteobacterium *Pseudomonas taeniospiralis* (Lalucat and Mayer 1978), now known as *Hydrogenophaga taeniospiralis*; the soil β-proteobacterium *Pseudomonas avenae* (Wells and Horne 1983), now known as *Acidovorax avenae* subsp. *avenae*; the soil strain *Pseudomonas* sp. EPS-5028 (Fusté *et al.* 1986); the anoxigenic photosynthetic N2-fixing α-proteobacterium *Rhodospirillum centenum* (Favinger *et al.* 1989); the soil strain *Pseudomonas aeruginosa* 44T1 (Espuny *et al.* 1991); and the melanin-producing marine γ-proteobacterium *Marinomonas mediterranea* (Hernandez-Romero *et al.* 2003). However, no further study on these R-body structures has been reported for any of these species [for review, see (Sanchez-Amat 2006)], nor have they been linked to the presence of Reb homologs in their genomes. Therefore, the role of these R-bodies in these diverse bacterial remains puzzling. A recent study has shown the presence of Reb homologs in the genome of the rhizobiale *Azorhizobium caulinodans*, a microsymbiont of the tropical legume *Sesbania rostrata* (Akiba *et al.* 2010). Interestingly, deletion of the putative transcription factor *praR* caused aberrant nodule formation and was linked to greater expression of the *reb* locus. On the contrary, a double *reb* and *praR* mutant had a restored wild-type nodule formation. The authors hypothesized that *praR* is essential to suppress the killer trait conferred by the *reb* locus and establish symbiosis between *A. caulinodans* and *S. rostrata* (Akiba *et al.* 2010). However, it is not known whether *A. caulinodans* is able to make R-bodies. The authors also reported the presence of Reb homologs in a number of Proteobacteria and in the Bacteroidetes member *Kordia algicida* OT-1 (Akiba *et al.* 2010).

Here, we have performed an exhaustive phylogenomic analysis of Reb homologs in currently available bacterial genomes. Reb homologs are widely distributed in members of *Proteobacteria*, comprising many free-living taxa as well as symbionts or pathogens of various eukaryotes, including humans. The evolutionary history of *reb* genes appears very dynamic, involving vertical inheritance, horizontal gene transfers, and gene duplications. By combining phylogenetic, genome synteny, and genomic content analyses, we highlight a few potential candidate partners of Reb proteins. Finally, we found no clear signs of *reb* loci originating from defective prophages, or from recent transfer via mobile elements. Ecological implications are discussed.

## Materials and Methods

### Homology searches

Reb proteins (A-D) encoded in the plasmid pKAP298 from *C. taeniospiralis* (AAR87077.1, AAR87076.1, AAR87131.1, AAR87075.1) were used as seeds to search for Reb homologs in the nonredundant protein database at the National Center for Biotechnology Information (NCBI). Homology searches were performed by BlastP (Altschul *et al.* 1997) and all hits within an *e*-value cutoff of 1 × 10^−3^ were retained. PSI-BLAST and tBLASTn programs (Altschul *et al.* 1997) also were used to search for highly divergent or misannotated Reb homologs. Searches were reiterated by using a number of seeds from various taxa. We also performed targeted searches against the metagenome and the viral sequence databases at the NCBI, and against all eukaryotic genome sequence available at the Joint Genome Institute (http://genome.jgi.gov). Sequences were aligned using Muscle 3.8.31 (Edgar 2004). Poorly aligned or divergent sequences were manually removed. Finally, HMM searches were performed with HMMER 3.0 (hmmer.org) against a local databank of 841 complete bacterial genomes (only one representative per species), including 435 from Proteobacteria downloaded from the NCBI ftp Genomes server using a model built on the multiple alignment of all previously recovered Reb proteins, but no additional homologs were found.

### Sequence analysis

Sequence secondary structures were predicted using PSIPRED (http://bioinf.cs.ucl.ac.uk/psipred/) (Buchan *et al.* 2010). Alpha helical wheel diagrams were created using the tool created by Don Armstrong and Raphael Zidovetzki (http://rzlab.ucr.edu/scripts/wheel/wheel.cgi). PredictProtein was used to search for additional structural features (http://www.predictprotein.org/). We searched for Reb homologs with available 3D structures using sequence based-PSI-BLAST (Altschul *et al.* 1997) searches at the PFAM and Uniprot databases by using HHpred (http://hhpred.tuebingen.mpg.de/hhpred) and FFAS03 (http://ffas.ljcrf.edu/ffas-cgi/cgi/ffas.pl). WebLogo analysis was performed at http://weblogo.berkeley.edu/.

### Phylogenetic analysis

The final dataset of 203 Reb homologs was aligned using Muscle 3.8.31 (Edgar 2004) and trimmed using BMGE (Criscuolo and Gribaldo 2010) with the less-stringent parameters (Blosum30), giving a dataset of 73 unambiguously aligned amino acid positions for phylogenetic analysis. A Bayesian tree was obtained using Phylobayes 3.3 (Lartillot *et al.* 2009). (See figure legends for details on analyses.)

16s rRNA sequences from Proteobacterial taxa representative of the diversity of this phylum were downloaded from the NCBI, as well as from the specialized Silva (http://www.arb-silva.de/) and the Ribosomal Database Project (http://rdp.cme.msu.edu/) databases. Sequences were aligned using Muscle 3.8.31 (Edgar 2004) and manually trimmed using the ED program of the MUST suite (Philippe 1993). Maximum likelihood trees were obtained using Treefinder (Jobb *et al.* 2004). (See figure legends for details on analyses.)

### Genome synteny and genome content analysis

For genome synteny analysis, we retrieved the five open reading frames upstream and downstream of the *reb* loci (or extracted from contigs when the whole genome sequence was not available). Protein sequences were defined as homologous when sharing at least 40% similarity and less than 20% difference in length. Pairs of homologous proteins were then expanded to homologous protein families by including all proteins homologous to at least one member of the family.

For genome content analysis, families of homologous proteins were built from 861 fully sequenced bacterial genomes downloaded from the NCBI ftp Genomes server. Protein sequences were defined as homologous if sharing at least 50% similarity and less than 20% difference in length. Pairs of homologous proteins were then expanded to homologous protein families by including all proteins homologous to at least one member of the family.

## Results

### Taxonomic distribution

**Figure 2:**
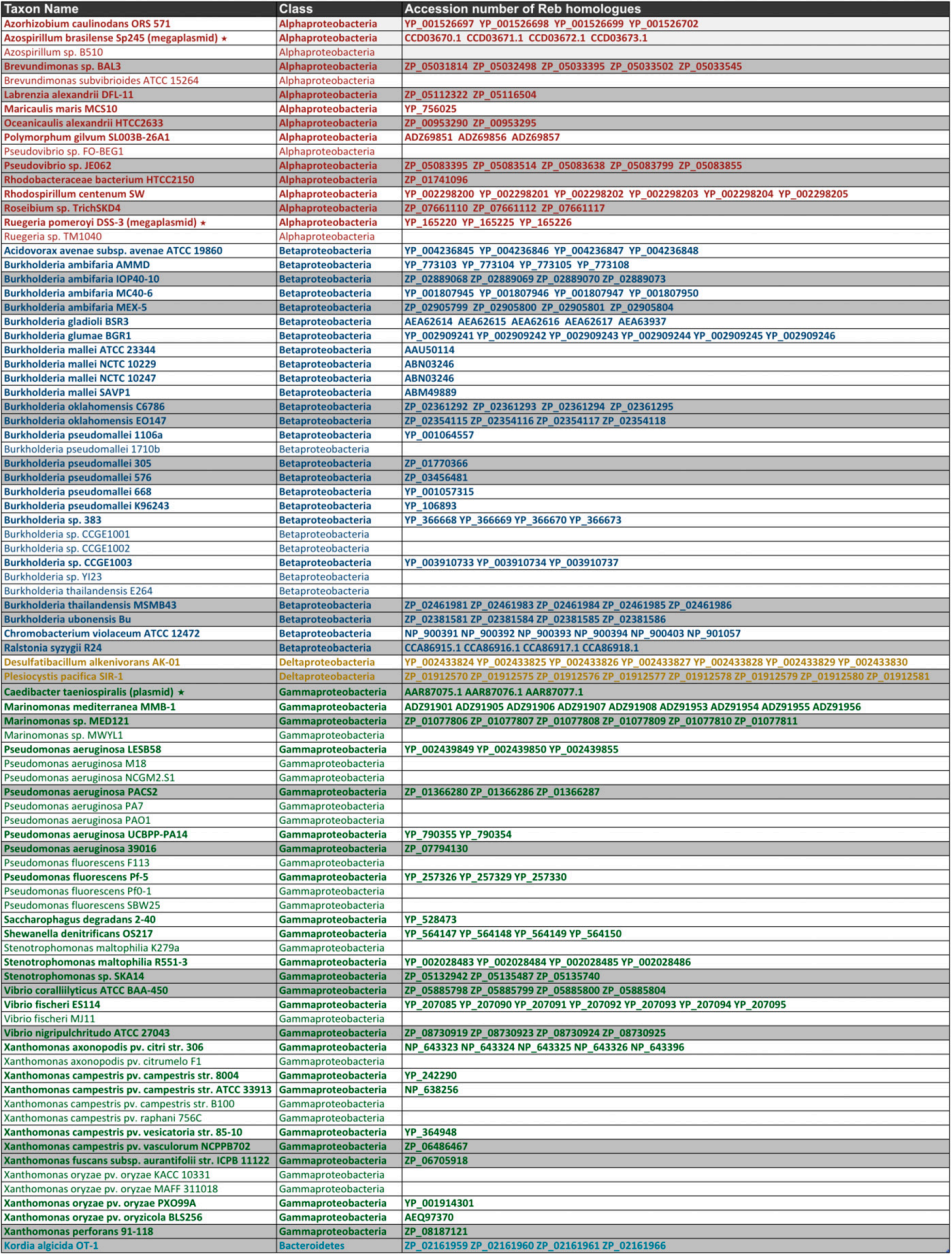
Distribution of Reb homologs. Presence/absence of Reb homologs in proteobacteria and *Kordia algicida*. Colors indicate the different proteobacterial subdivisions. For each genome that harbors Reb homologs, we included complete genomes of closely related taxa without any *reb* genes, when available. Taxa with no available complete genome sequence but harboring Reb homologs are highlighted in gray. For these taxa, the presence of extra Reb copies cannot be excluded. When present, Reb homologs are indicated by their corresponding accession number. Reb homologs located on plasmids are indicated by an asterisk. See main text for discussion.

**Figure 3:**
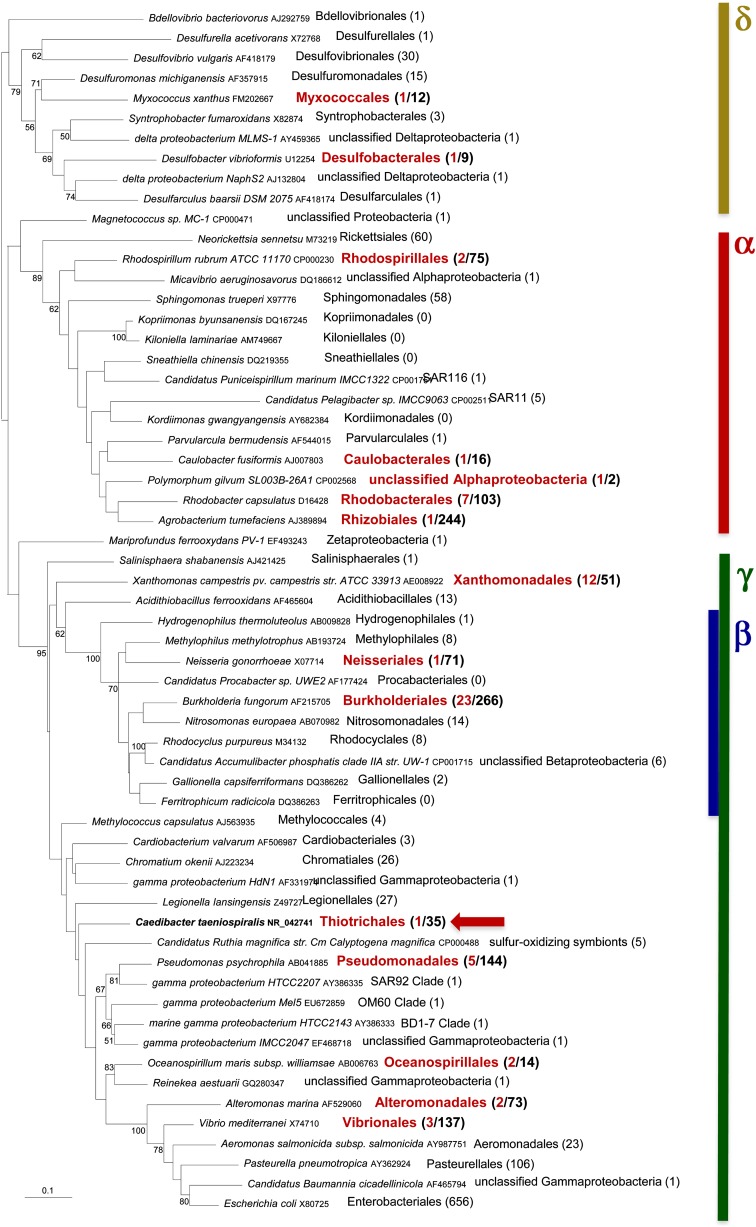
Distribution of Reb-harboring taxa across Proteobacteria. Unrooted Maximum likelihood phylogenetic tree of 16s rRNA sequences from 60 taxa representative of proteobacterial diversity. Proteobacterial orders that include members containing Reb homologs are highlighted in red. The number of Reb-harboring taxa over the total number of available complete genomes is indicated in parenthesis. *Caedibacter taeniospiralis* belongs to the gammaproteobacterial family of Thiotrichales (indicated by a red arrow). The tree was obtained using Treefinder with the J1 model of nucleotide substitution and a discrete gamma distribution with four categories to take into account among-site rate variation. Numbers at nodes indicate bootstrap values (BV) for 100 replicates of the original dataset. For clarity, only BVs greater than 50% are shown. The scale bar represents the average number of substitutions per site.

Although the production of R-bodies has been observed in a few free-living bacteria (see Introduction), the effective distribution of Reb homologs in prokaryotes has not been clear. We carried out an exhaustive search for Reb homologs in current sequence databases (see Materials and Methods) (Figure 2). We found no additional homologs of RebC other than the *C. taeniospiralis* pKAP298 plasmid, indicating that this protein is specific to the Caedibacter Reb system. On the contrary, RebB, RebA and RebD are homologous and widely distributed. We identified 203 Reb homologs from 64 taxa belonging exclusively to *Proteobacteria*, with the one exception of *Kordia algicida* OT-1, which belongs to the phylum *Bacteroidetes*, as recently noticed (Akiba *et al.* 2010). Reb homologs are widely distributed among representatives of four of the six subdivisions of *Proteobacteria*, Alpha, Beta, Gamma, and Delta (Figure 3). We found between one and nine Reb homologs in each genome (Figure 2). Although *reb* genes were first identified on the *C. taeniospiralis* pKAP298 plasmid, we found Reb homologs on only two additional plasmids: the megaplasmid from the α-proteobacterium *Ruegeria pomeroyi* DSS-3, and the AZOBR_p4 plasmid from the α-proteobacterium *Azospirillum brazilense* Sp245 (Figure 2). The availability of a complete genome for these two taxa indicates that no additional homologs are present on the chromosome. We could not find any other Reb homologs in viruses, Archaea, or Eukarya, apart from four homologs from the Global Ocean Sampling marine metagenome sequence database that are closely related to Proteobacteria (not shown).

Of importance, for three taxa with available sequence data, we could link for the first time the previously reported observation of R-bodies (see *Introduction* and references therein) with the presence of Reb homologs. In particular, *R. centenum* has six copies, *A. avenae* has four copies, and *M. mediterranea* has nine copies (Figure 2). Reb-containing taxa display a wide variety of lifestyles. Albeit many taxa harboring Reb homologs harbor free-living lifestyles in a wide variety of environments, from marine to terrestrial, a few taxa other than *C. taeniospiralis* appear to have an interaction with eukaryotes. For example, *Pseudovibrio sp. JE062* is a symbiont of sponges; *Vibrio fischeri ES114* is the specific bioluminescent symbiont in the light-emitting organs of certain squids and fishes; *Labrenzia alexandrii* and *Oceanicaulis alexandrii* have been isolated from dinoflagellates; *Stenotrophomonas maltophilia* R551-3, *Azospirillum brasilense* Sp245, and *Pseudomonas fluorescens* Pf-5 are plant growth−promoting endophytes; various strains of *Burkholderia ambifaria* are important in the biocontrol of pea plant phytopathogens; and *Azorhizobium caulinodans* is a nitrogen-fixing proteobacterium involved in mutualistic rhizobiale symbioses with plant roots.

Other than in the algae pathogen *Kordia algicida*, we also found Reb homologs in a number of important pathogens of plants such as *Acidovorax avenae subsp. avenae* ATCC 19860; various strains of *Xanthomonas*; *Burkholderia gladioli and B. glumae*; *Ralstonia syzygii* R24; but also in important pathogens of aquatic animals such as shrimp and corals (*Vibrio nigripulchritudo* ATCC27043; *Vibrio coralliilyticus* ATCC-BAA450). Reb homologs were also found in the genomes of opportunistic pathogens of humans, such as various strains of *Pseudomonas aeruginosa*, including the hypervirulent LESB58 strain; in various strains of *Burkholderia pseudomallei*, the causative agent of meilodiosis, in *B. mallei*, that causes glanders and pneumonia; and *Stenotrophomonas maltophila*, a rare but serious threat to patients who require catheterization. We also observed some interesting patterns by looking at the distribution of Reb homologs in closely related strains (Figure 2). For example, although the sponge symbiont *Pseudovibrio sp*. JE062 harbors *reb* genes, its closely related free-living relative *Pseudovibrio sp*. FO-BEG1 does not. Similarly, *Burkholderia thailandensis* MSMB43, an opportunistic pathogen that causes meilodiosis, has *reb* genes, whereas the closely related *B. thailandensis* E264, a common soil and avirulent strain, does not (Figure 2).

### Sequence analysis

Despite the widespread presence of Reb homologs in many bacterial taxa, it remains to be proven experimentally that these are responsible for producing R-bodies. However, some hints can already be gained from sequence analysis.

**Figure 4:**
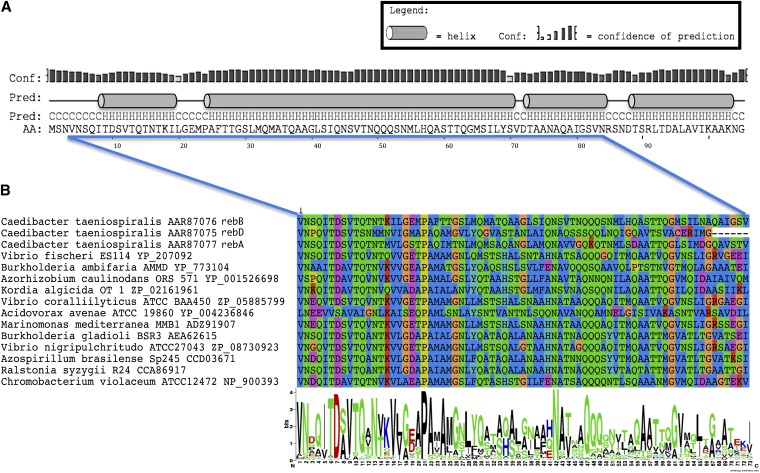
Sequence analysis. (A) Secondary structure of the RebB of *C. taeniospiralis* predicted by PSIPRED [http://bioinf.ucl.ac.uk/psipred (Buchan *et al.* 2010)]. The same structure was substantially conserved in all other Reb homologs. (B) Conserved amino acid positions identified using Weblogo on an unambiguously aligned excerpt of the entire alignment of the 203 identified Reb homologs. For clarity, only 15 representative Reb sequences are shown. Position numbers refer to the RebB of *C. taeniospiralis*.

Reb homologs are 95 amino acids long on average. They show good conservation at the sequence level and all display a basic alpha helical secondary structure with no significant structural difference among sequences (Figure 4A). Very little is known about the regulation and mechanism of R-body assembly [for review see, (Sanchez-Amat 2006)]. We could not observe any particular pattern in the sequences that allows distinguishing the equivalents of RebA, B, and D of *C. taeniospiralis* in other taxa, and this was also confirmed by phylogenetic analysis. Overall sequence conservation is high, suggesting that these Reb homologs are likely functional and conserved at the structural level. A WebLogo analysis highlighted highly conserved positions (Figure 4B) that may be important for R-body assembly and/or unrolling-rolling, and should be the target of choice of future mutation studies. Unfortunately, we could not identify any homologous proteins with a solved crystal structure in extant databases (see *Materials and Methods*). Small proteins assembling into structures frequently display amphipathic helices, which consist of hydrophobic amino acids concentrated on one side and hydrophilic or polar amino acids on the other, and these can be highlighted using helical wheel diagrams (see *Materials and Methods*). However, we found no evidence that the helices of Reb homologs display amphipathic character (data not shown). Obtaining the 3-D structure of an R-body will therefore be essential to understand how Reb homologs assemble and function.

**Figure 5:**
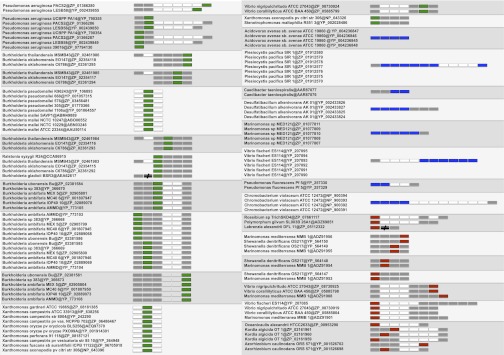
Evolutionary inference of Reb homologs based on phylogenetic analysis of the 203 Reb homologs (Figure S1). Here we have highlighted a few of the monophyletic groups. For each taxon, the genome locations of the corresponding Reb proteins are shown. Reb homologs highlighted in green are orthologs that were inferred to have been inherited through speciation events; those highlighted in blue represent paralogs issued from species-specific gene duplications; and those in red are the Reb homologs that have likely originated via horizontal transfer. Adjacent *reb* genes are indicated in gray. Open reading frames between *reb* genes are shown in white, and black slash-like symbols represent large intervening regions between *reb* genes.

When present in multiple copies, Reb homologs are clustered on the genome, mostly lying side-by-side or separated by a few intervening genes (Figure 5). We generally found only one *reb* locus per genome, with the exception of *M. mediterranea* whose nine homologs are organized into two different genomic regions, and *Xanthomonas axonopodis* and *Chromobacterium violaceum*, which both have an extra Reb homolog located far from the main cluster (Figure 5). Given their short length, the phylogeny of Reb homologs is globally poorly resolved, but a few monophyletic groups are apparent which are consistent with genomic synteny (Supporting Information, Figure S1 and Figure 5). This allowed us to infer the evolutionary history of these Reb homologs (Figure 5). In some cases, Reb homologs from the same taxon are more closely related to each other than to Rebs of other taxa, suggesting that these have arisen from species-specific duplication events.

This is for, instance, the case of five *reb* genes from *Marinomonas sp*. MED121, which are all more closely related to each other than to any other Reb (Figure 5). The same can be said for the six of the seven *reb* genes from *Vibrio fischeri* ES114 (Figure 5). In other cases, there is clear evidence for vertical inheritance of Reb proteins from the ancestor of a specific *Proteobacterial* family (*e.g.*, *Xanthomonas*) (Figure 5). In yet other instances, Reb proteins are most closely related among distantly related lineages, suggesting horizontal transfer of the whole locus, for example in the case of Marinomonas mediterranea MMB-1 and Shewanella denitrificans OS217 (Figure 5). Horizontal gene transfer was also suggested for extra *reb* copies in a few taxa (Figure 5). Finally, phylogeny could not help in assigning the equivalents in other taxa of the RebA-B-D of *C. taeniospiralis*, as these are more closely related among themselves (Figure S1). It is therefore difficult to make analogies between the previously reported data on the role of the RebA-B-D of *C. taeniospiralis* in the assembly process of its R-bodies and what occurs in the other taxa. Moreover, because we found no homologs of RebC outside *C. taeniospiralis*, it is possible that other proteins have analogous function in Reb-harboring taxa. This finding would be consistent with RebC being a transcription regulator and therefore potentially species-specific.

### In search for potential partners of Reb proteins

It has been shown that RebA, B and C from *C. taeniospiralis* are sufficient for production of type 51 R-body in *E. coli* but not for the killing phenotype (see *Introduction*), which indicates that yet-unidentified partners coded on either the plasmid or the chromosome of *C. taeniospiralis* are involved in the killing. Interestingly, we found no homologs of the 63 proteins encoded in the *C. taeniospiralis* pKAP298 plasmid in any of the Reb-harboring taxa. This may indicate that none of these proteins is a likely candidate for the killing toxin, which would be then encoded in the *C. taeniospiralis* genome (yet unavailable). Alternatively, the *C. taeniospiralis* toxin may well be on the plasmid but is not conserved in other bacteria, which may either display no killing activity or use nonhomologous toxins.

**Figure 6:**
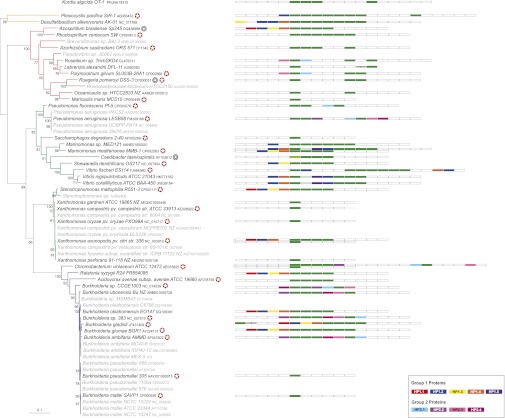
Genome synteny analysis of the *reb* locus mapped onto an Unrooted Maximum likelihood phylogenetic tree of 16s rRNA sequences from the 64 *reb*-containing Proteobacteria. The tree was created using Treefinder with the GTR model of nucleotide substitution and a discrete gamma distribution with four categories to take into account among-site rate variation. Numbers at nodes indicate BVs for 100 replicates of the original dataset. For clarity, only BVs greater than 50% are shown. The scale bar represents the average number of substitutions per site. Species where Reb homologs are located on a plasmid are marked by a black circle. A white star in a red circle marks the fully sequenced genomes used in the analysis. Reb homologs are shown in green. Homologous genes are represented by the same color. For clarity, only genes discussed in the text are indicated. Black slash-like symbols represent large regions in between genes. The RebC of *C.taeniospiralis* (AAR87131) is shown in light green and outlined in black to indicate its lack of homology with the other *rebs*. The genome context for the *Flavobacterium K. algicida* is shown separately.

To search for candidate partners of Reb proteins, we carried out a genome synteny analysis of the *reb* locus in 41 taxa for which a complete genome or sufficient genomic structure information is available (Figure 6, see *Materials and Methods*). Two mutually exclusive synteny patterns could be observed (hereafter referred to as Group 1 and 2, respectively; Figure 6). Strikingly, the genes included in these conserved synteny patterns are exclusively present in Reb-harboring taxa, strongly suggesting a functional link with the Reb system. The Group 1 synteny pattern is defined by four proteins annotated as hypothetical: HP1.1 (red), HP1.2 (blue), HP1.3 (yellow), and HP1.4 (orange), which are only found in the surroundings of the *reb* locus and are only present in Reb-harboring taxa. The HP1.4 (~60−80 aa) and HP1.3 (!170 aa) proteins appear to be distant Reb homologs. However, they lack some of the conserved amino acid positions characteristic of other Reb proteins, and the HP1.3 protein is approximately twice as long as a typical Reb (data not shown). The HP1.1 (~360 aa) and the HP1.2 (~110−120 aa) proteins display no putative conserved domains. These four proteins often exhibit the same genomic organization (HP1.1,HP1.2,HP1.3,HP1.4; Figure 6). In three cases, another hypothetical protein (HP1.5, purple) is associated with this context, and is distantly related to the HP1.3 protein (Figure 6).

The Group 2 synteny pattern is defined by the presence of two proteins annotated as hypothetical: HP2.1 (light blue) and HP2.2 (fuchsia) (Figure 6). These proteins are approximately the same size (~205−220 aa) and display no putative conserved domains. They frequently co-occur, but their genomic organization varies in different taxa. An interesting characteristic of Group 2 synteny pattern is the frequent association with a putative RNA polymerase sigma-factor protein (HP2.3, light pink) and a transcriptional regulator/cyclic nucleotide binding protein (HP2.4, dark pink), which might be involved in transcription regulation of *reb* genes in these taxa. It should be noted that the conserved Group 1 and Group 2 synteny patterns are generally consistent with the Reb clusters highlighted by phylogenetic analysis (Figure 5) and have phylogenies similar to the Reb one (not shown), indicating a common evolutionary history and providing further suggestion of a functional link between these proteins and Reb proteins. As an additional strong indication, the plasmid sequence of *A. brasilense* contains the four proteins characteristic of the Group 1 synteny pattern.

**Figure 7:**
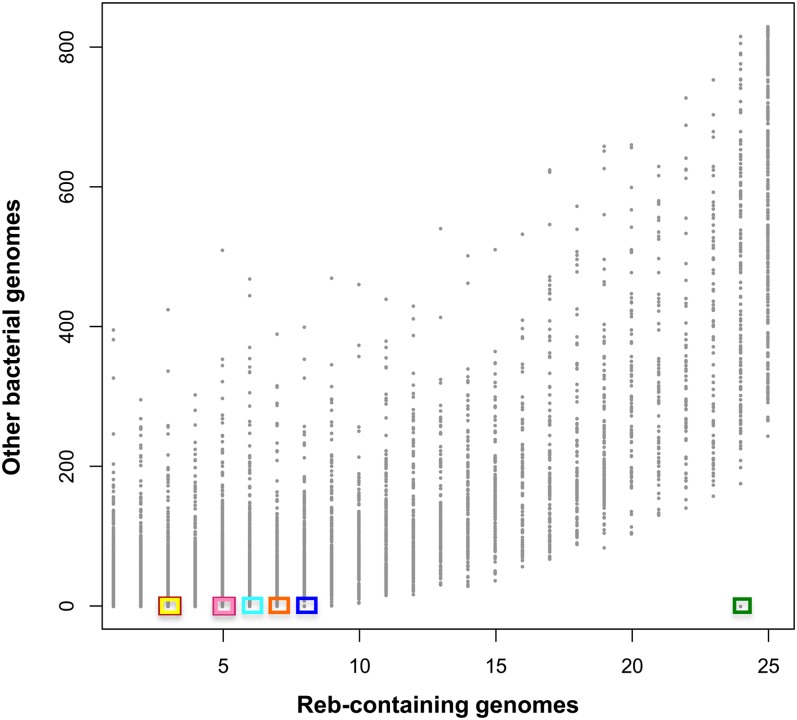
Whole genome content analysis. Graphical representation of protein families created using the R software (R Development Core Team 2011). The 25 fully sequenced Reb-harboring taxa are represented on the x-axis and the other 816 fully sequenced bacterial taxa analyzed are represented on the y-axis. Each point on the graph represents a protein family (see *Materials and Methods* for details on how protein families were defined). For example, the Reb family (indicated by a green box) is present in 24 fully sequenced Reb-harboring taxa but in none of the remaining genomes. The 4 Rebs of *Acidovorax avenae* subsp. Avenae ATCC 19860 did not fall into the Reb protein family because they are very divergent (see Figure 5 and Figure S1). The other five unique protein families specific to Reb-harboring taxa (see main text) are shown with boxes corresponding to colors as defined in the legend to Figure 6.

A few taxa did not present any particularly conserved genomic context nor did they harbor the conserved genes found in Group 1 or Group2 synteny patterns. It is therefore possible that other genetic elements important for *reb* function are located in different positions of the genome in these taxa. To this end, we sought to identify additional proteins specific to Reb-harboring taxa by carrying out a whole-genome content analysis (see *Material and Methods*). Using the complete genomes of 841 bacterial taxa—including 25 Reb-harboring complete genomes—we constructed protein families having at least 50% identity and 80% size conservation (see *Material and Methods*). This analysis confirmed that the only protein family exclusively present in Reb-harboring taxa is the Reb family itself, along with the protein families specific to Group 1 and 2 synteny patterns (Figure 7). Although genomes of taxa not containing Reb homologs harbored distant homologs of the HP2.3 and HP2.4 (light pink and dark pink) proteins of genome synteny patterns, these fell outside of subfamilies which are exclusively present in Reb-harboring taxa.

These few conserved proteins might be either involved in regulation of R-body assembly and function, or represent the toxin, and should be priority targets for future studies. It will also be interesting to test whether the Reb-harboring taxa that harbor none of these candidate partners are able to make R-bodies or display killing activity. Finally, it should be noted that none of these proteins belong to the Soj-ParA family or any annotated membrane-associated ATPase, weakening the previous hypothesis that these types of protein may represent the toxin responsible for killing (Jeblick and Kusch 2005).

### A phage origin?

Jeblick and Kush emphasized the presence of phage-related genes on the *reb* carrying plasmid (Jeblick and Kusch 2005) and Preer (Preer *et al.* 1974) observed an association of phage-like particles with R-bodies, suggesting that R-bodies may be encoded by defective phage genes. Moreover, the evolutionary analysis of Reb families (Figure 5) and their genomic context (Figure 6) suggest horizontal gene transfer events, for which bacteriophages are known to be major contributors. However, we found no Reb homologs in genomic sequences from phages. We therefore sought to see if Reb homologs are part of integrated elements or prophages. We examined the 40 kbp on each side of the *reb* locus in the 25 Reb-harboring taxa for which complete genome sequences are available for the presence of prophages (integrated phages) or other phage-related elements. First, we searched using the PHAST database [http://phast.wishartlab.com (Zhou *et al.* 2011)], which contains phage proteins that have been associated with a clear phage function. However, none of these regions were positive for prophage sequences. As a complementary analysis, we specifically searched a comprehensive local databank of 1130 bacteriophage sequences downloaded from GenBank (December 2011), which included 248 phages isolated from 33 proteobacterial genera. These regions did not display any specific similarity to phage elements. We also looked at whether *reb* loci are embedded in genomic islands by running searches on the IslandViewer server [http://www.pathogenomics.sfu.ca/islandviewer (Langille and Brinkman 2009)], which combines several prediction methods: (1) atypical dinucleotide content; or (2) codon usage; (3) identification of unique regions not present in closely related genomes; and (4) presence of genes that are functionally related to mobile elements. However, none of the analyzed genomes displayed identified potential genomic islands adjacent to or surrounding the *reb* locus. We also verified from the literature whether Reb homologs were present in any previously reported genomic region of potential exogenous origin. For example, *reb* genes did not fall into any of the two atypical regions identified in the genome of *Xanthomonas oryzae pv. oryzae* PXO99A (Salzberg *et al.* 2008), nor in the four atypical regions highlighted in the genome of *Xanthomonas campestris pv. campestris* str. ATCC 33913 (Vorholter *et al.* 2003), and were not included in any of the prophage islands identified in the genome of the *Pseudomonas aeruginosa* hypervirulent LESB58I strain (Winstanley *et al.* 2009). Finally, among 1062 plasmid sequences available from *Proteobacteria*, we detected *reb* genes only on two plasmids other than the *C. taeniospiralis* plasmid pKAP298: the *Azospirillum brasilense* Sp245 plasmid AZOBR_p4 and the *Ruegeria pomeroyi* DSS-3 megaplasmid.

## Discussion

Despite having been continuously and intensively studied from the 1930s through the 1980s, recent data on R-bodies have been scarce. With a whole array of novel technology, studies on the diversity and role of these puzzling bacterial structures can now be fully tackled. Our exhaustive analysis shows that Reb homologs are widely present in Proteobacterial genomes spanning the diversity of this major bacterial phylum, indicating that they are much more widespread than previously known. In the perspective of obtaining experimental data, our analysis remains for the time being largely descriptive, but nevertheless provides a number of interesting hints for discussion and future work.

Sequence analysis suggests structural and functional conservation, indicating that Reb homologs are likely responsible for the production of functional R-bodies in all the taxa where we found them, although this needs to be verified experimentally. Moreover, the presence of Reb homologs in bacteria where R-bodies have been previously observed is already a good hint. The occurrence of R-bodies in a wide range of bacteria harboring Reb homologs should be tested, with priority given to those that have medical, agricultural, and ecological implications. Our data will also help direct mutational studies to characterize the system further through structural and functional analysis of *reb* genes from Caedibacter but also other taxa, including those that harbor multiple *reb* genes and those that have only one copy. Also, it will be important to verify the involvement in R-body production, assembly, regulation, and killing of the likely partners that we have identified by genome context and whole genome content analysis. Because none of the Reb-harboring genomes identified in this study possess homologs of the genes carried by the *C. taeniospiralis* plasmid, it is possible that the killing toxin is encoded in the *C. taeniospiralis* genome and is perhaps one of the candidate genes that we have identified. The completion of this genome will therefore be very important. Another possibility is that the R-bodies are delivery systems for species-specific toxins.

Our analysis of taxonomic distribution shows that Reb homologs are present in taxa displaying very different lifestyles, suggesting that the role of R-bodies in nature could be quite diverse. Moreover, we show that intact *reb* loci have been spread among Proteobacterial taxa via horizontal transfer, indicating that an advantage exists in acquiring and keeping R-bodies. However, we found no clear signs of a phage origin for the *reb* loci. It remains possible that this is due to an undersampling of phages from the Reb-harboring taxa or that these horizontal transfers correspond to events old enough to have allowed sequences to adapt to the new genome, or that all traces of the transfer vectors have been erased from the genome after transfer.

Some of the Reb-harboring taxa have very important ecological, agricultural, and medical relevance. In addition, by observing the pattern of presence/absence of *reb* genes in closely related strains, we found intriguing links between virulence and presence of *reb* genes that will surely be worthy of further investigation. R-bodies may be involved in mediating interactions of these bacteria with eukaryotic cells, perhaps through the triggering of unrolling when ingested in the vacuole, similarly to what observed in the Caedibacter/Paramecium interaction.

We found Reb homologs in many free-living bacteria. R-bodies in these bacteria may be used as a defense mechanism against grazing by eukaryotes. Bacteria have in fact developed various strategies against protozoan predation in nature (Matz and Kjelleberg 2005). Many examples have been reported of cytotoxicity responses against grazing by eukaryotes in different bacteria such as *Pseudomonas* (Matz *et al.* 2004; Weitere *et al.* 2005) and *Vibrio* (Erken *et al.* 2011). Also, it is tempting to speculate that some of these free-living, Reb-harboring taxa can establish transient “killing” symbioses with ciliates or other protists, as seems to be indicated by the fact that some Reb-harboring taxa have indeed been isolated from aquatic microbial eukaryotes. It will be interesting to perform feeding experiments to test their killing potential in Paramecium and also other protistan taxa, such as algae. Indeed, we confirmed the presence of *reb* genes in *Kordia algicida*, a planktonic bacterium recently highlighted as a killer of diatoms by a yet unclear mechanism involving an unidentified protease (Paul and Pohnert 2011). Our study suggests that delivery of this protease could be performed via R-bodies, which would therefore be important players in the regulation of algal blooms. Similarly, it would be interesting to verify whether the presence of Reb homologs in the powerful coral pathogen *Vibrio coralliilyticus* is linked to R-body production and if these are somehow involved in delivery of the killing toxin. The killing factor produced by *K. algicida* is triggered independently of the presence of the diatom target but rather likely depends on a quorum sensing mechanism when the population size reaches a certain density (Paul and Pohnert 2011). Similarly, the triggering of R-body production in a fraction of the Caedibacter endosymbiont population in Paramecium, a phenomenon that is not yet understood, may be regulated by a quorum sensing mechanism.

In addition, our study suggests that R-bodies may be involved in the interaction of Proteobacteria with several multicellular organisms, such as plants and animals. The recently reported involvement of *reb* genes in the regulation of a rhizobial symbiosis (Akiba *et al.* 2010) and the presence of Reb homologs in a number of Proteobacteria known to interact with plant roots is intriguing, and it is not excluded that R-bodies may help the bacterium to move through plant tissues, via delivery of specific lytic compounds. Indeed, we found Reb homologs in a number of bacterial strains known to be able to penetrate the xylem of plants. A similar mechanism may be used to move through tissues by some Proteobacteria that interact with animals, such as *Vibrio fischeri* with its squid host. Finally, the presence of *reb* genes in important pathogens of eukaryotes, including humans, some of which are responsible for emerging and poorly characterized infections, should prompt the study of their potential involvement in the infection process, perhaps by helping tissue invasion.

If our predictions are verified, bacteria may represent a largely overlooked role in the regulation of microbial eukaryotic abundance and distribution, in addition to the much more studied impact of viruses. This regulation may be performed at different levels, by direct killing of eukaryotic grazers, but also by providing mechanisms used for defense among eukaryotes, as is the example of the Paramecium/Caedibacter symbiosis. Interestingly, it was recently reported that the thricocysts of eukaryotic algae belonging to the Cryptomonads, ejectile organelles that are probably used with a defensive role against predation, are composed of four proteins that share similarity with Reb proteins (Yamagishi *et al.* 2012). The authors proposed that these proteins were acquired horizontally from Proteobacteria. R-body−harboring bacteria could therefore play a larger role in the origin and spread of defense mechanisms in eukaryotic microorganisms. Finally, elucidation of the mechanism of rolling/unrolling/toxin delivery of R-bodies will surely open the way to interesting biotechnological applications.

## Supplementary Material

Supporting Information
