# Supplementary material for: A Genomic Survey of Reb Homologs Suggests Widespread Occurrence of R-Bodies in Proteobacteria
Source: G3 (Bethesda). 2013 Mar 1;3(3):505–16. doi: 10.1534/g3.112.005231 (PMC3583457; doi:10.1534/g3.112.005231)
Supplement: Supporting Information [file supp_3_3_505__index.html]

Supporting Information 

# A Genomic Survey of Reb Homologs Suggests Widespread Occurrence of R-Bodies in Proteobacteria

## Supporting Information for Raymann *et al.*, 2013

**Files in this Data Supplement:**

- Figure S1 - Phylogeny of Reb homologues (PDF, 370 KB)
