# Supplementary material for: A Genomic Survey of Reb Homologs Suggests Widespread Occurrence of R-Bodies in Proteobacteria
Source: G3 (Bethesda). 2013 Mar 1;3(3):505–16. doi: 10.1534/g3.112.005231 (PMC3583457; doi:10.1534/g3.112.005231)
Supplement: Supporting Information [file supp_3.3.505_FigureS1.pdf]

G3 · Genes | Genomes | Genetics

A genomic survey of Reb homologues suggests widespread occurrence of R-bodies in Proteobacteria

Kasie Raymann[*,§], Louis-Marie Bobay [§,**,§§], Thomas G. Doak[***], Michael Lynch[***], Simonetta Gribaldo[*,1]

[*] Institut Pasteur, Unité Biologie Moleculare du Gene chez les Extremophiles, Departement de Microbiologie, Paris, 75724 Cedex 15 France
[§] Univ. Pierre et Marie Curie, Cellule Pasteur UPMC, Paris, 75724 Cedex 15 France
[**] Institut Pasteur, Microbial Evolutionary Genomics, Departement Genomes et Genetique, Paris, 75724 Cedex 15 France
[§§] CNRS, UMR3525, Paris, F-75015 France
[***] Indiana University, Department of Biology, 1001 East Third St., Bloomington, Indiana 47405

Corresponding author:
Simonetta Gribaldo
Unité Biologie Moleculare du Gene chez les Extremophiles, Departement de Microbiologie
Institut Pasteur
25-28 rue du Dr Roux 75724
Paris Cedex 15, France
E-mail: simonetta.gribaldo@pasteur.fr
Office Phone: +33144389455
Fax: +33145688834

DOI: 10.1534/g3.112.005231

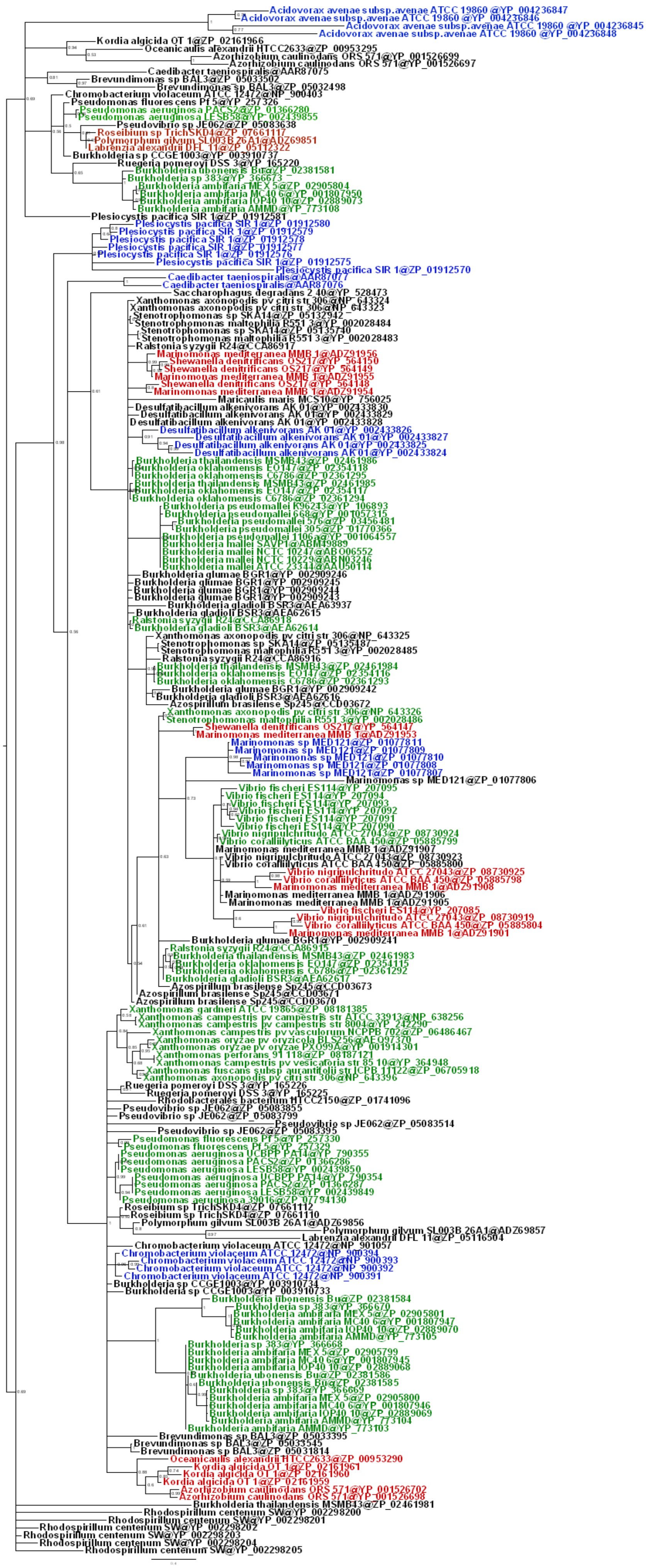

Acidovorax avenae subsp.avenae ATCC 19860 @YP_004236847
Acidovorax avenae subsp.avenae ATCC 19860 @YP_004236846
Acidovorax avenae subsp.avenae ATCC 19860 @YP_004236845
Acidovorax avenae subsp.avenae ATCC 19860 @YP_004236848
Kordia algicida OT 1@ZP_02161966
Oceanicaulis alexandrii HTCC2633@ZP_00953295
Azorhizobium caulinodans ORS 571@YP_001526699
Azorhizobium caulinodans ORS 571@YP_001526697
Caedibacter taeniospiralis@AAR87075
Brevundimonas sp BAL3@ZP_05033502
Brevundimonas sp BAL3@ZP_05032498
Chromobacterium violaceum ATCC 12472@NP_900403
Pseudomonas fluorescens Pf 5@YP_257326
Pseudomonas aeruginosa PACS2@ZP_01366280
Pseudomonas aeruginosa LESB58@YP_002439855
Pseudovibrio sp JE062@ZP_05083638
Roseibium sp TrichSKD4@ZP_07661117
Polymorphum gilvum SL003B 26A1@ADZ69851
Labrenzia alexandrii DFL 11@ZP_05112322
Burkholderia sp CCGE1003@YP_003910737
Ruegeria pomeroyi DSS 3@YP_165220
Burkholderia ubonensis Bu@ZP_02381581
Burkholderia sp 383@YP_366673
Burkholderia ambifaria MEX 5@ZP_02905804
Burkholderia ambifaria MC40 6@YP_001807950
Burkholderia ambifaria IOP40 10@ZP_02889073
Burkholderia ambifaria AMMD@YP_773108
Plesiocystis pacifica SIR 1@ZP_01912581
Plesiocystis pacifica SIR 1@ZP_01912580
Plesiocystis pacifica SIR 1@ZP_01912579
Plesiocystis pacifica SIR 1@ZP_01912578
Plesiocystis pacifica SIR 1@ZP_01912577
Plesiocystis pacifica SIR 1@ZP_01912576
Plesiocystis pacifica SIR 1@ZP_01912575
Plesiocystis pacifica SIR 1@ZP_01912570
Caedibacter taeniospiralis@AAR87077
Caedibacter taeniospiralis@AAR87076
Saccharophagus degradans 2 40@YP_528473
Xanthomonas axonopodis pv citri str 306@NP_643324
Xanthomonas axonopodis pv citri str 306@NP_643323
Stenotrophomonas sp SKA14@ZP_05132942
Stenotrophomonas maltophilia R551 3@YP_002028484
Stenotrophomonas sp SKA14@ZP_05135740
Stenotrophomonas maltophilia R551 3@YP_002028483
Ralstonia syzygii R24@CCA86917
Marinomonas mediterranea MMB 1@ADZ91956
Shewanella denitrificans OS217@YP_564150
Shewanella denitrificans OS217@YP_564149
Marinomonas mediterranea MMB 1@ADZ91955
Shewanella denitrificans OS217@YP_564148
Marinomonas mediterranea MMB 1@ADZ91954
Maricaulis maris MCS10@YP_756025
Desulfatibacillum alkenivorans AK 01@YP_002433830
Desulfatibacillum alkenivorans AK 01@YP_002433829
Desulfatibacillum alkenivorans AK 01@YP_002433828
Desulfatibacillum alkenivorans AK 01@YP_002433826
Desulfatibacillum alkenivorans AK 01@YP_002433827
Desulfatibacillum alkenivorans AK 01@YP_002433825
Desulfatibacillum alkenivorans AK 01@YP_002433824
Burkholderia thailandensis MSMB43@ZP_02461986
Burkholderia oklahomensis EO147@ZP_02354118
Burkholderia oklahomensis C6786@ZP_02361295
Burkholderia thailandensis MSMB43@ZP_02461985
Burkholderia oklahomensis EO147@ZP_02354117
Burkholderia oklahomensis C6786@ZP_02361294
Burkholderia pseudomallei K96243@YP_106893
Burkholderia pseudomallei 668@YP_001057315
Burkholderia pseudomallei 576@ZP_03456481
Burkholderia pseudomallei 305@ZP_01770366
Burkholderia pseudomallei 1106a@YP_001064557
Burkholderia mallei SAVP1@ABM49889
Burkholderia mallei NCTC 10247@ABO06552
Burkholderia mallei NCTC 10229@ABN03246
Burkholderia mallei ATCC 23344@AAU50114
Burkholderia glumae BGR1@YP_002909246
Burkholderia glumae BGR1@YP_002909245
Burkholderia glumae BGR1@YP_002909244
Burkholderia glumae BGR1@YP_002909243
Burkholderia gladioli BSR3@AEA63937
Burkholderia gladioli BSR3@AEA62615
Ralstonia syzygii R24@CCA86918
Burkholderia gladioli BSR3@AEA62614
Xanthomonas axonopodis pv citri str 306@NP_643325
Stenotrophomonas sp SKA14@ZP_05135487
Stenotrophomonas maltophilia R551 3@YP_002028485
Ralstonia syzygii R24@CCA86916
Burkholderia thailandensis MSMB43@ZP_02461984
Burkholderia oklahomensis EO147@ZP_02354116
Burkholderia oklahomensis C6786@ZP_02361293
Burkholderia glumae BGR1@YP_002909242
Burkholderia gladioli BSR3@AEA62616
Azospirillum brasilense Sp245@CCD03672
Xanthomonas axonopodis pv citri str 306@NP_643326
Stenotrophomonas maltophilia R551 3@YP_002028486
Shewanella denitrificans OS217@YP_564147
Marinomonas mediterranea MMB 1@ADZ91953
Marinomonas sp MED121@ZP_01077811
Marinomonas sp MED121@ZP_01077809
Marinomonas sp MED121@ZP_01077810
Marinomonas sp MED121@ZP_01077808
Marinomonas sp MED121@ZP_01077807
Marinomonas sp MED121@ZP_01077806
Vibrio fischeri ES114@YP_207095
Vibrio fischeri ES114@YP_207094
Vibrio fischeri ES114@YP_207093
Vibrio fischeri ES114@YP_207092
Vibrio fischeri ES114@YP_207091
Vibrio fischeri ES114@YP_207090
Vibrio nigripulchritudo ATCC 27043@ZP_08730924
Vibrio coralliilyticus ATCC BAA 450@ZP_05885799
Marinomonas mediterranea MMB 1@ADZ91907
Vibrio nigripulchritudo ATCC 27043@ZP_08730923
Vibrio coralliilyticus ATCC BAA 450@ZP_05885800
Vibrio nigripulchritudo ATCC 27043@ZP_08730925
Vibrio coralliilyticus ATCC BAA 450@ZP_05885798
Marinomonas mediterranea MMB 1@ADZ91908
Marinomonas mediterranea MMB 1@ADZ91906
Marinomonas mediterranea MMB 1@ADZ91905
Vibrio fischeri ES114@YP_207085
Vibrio nigripulchritudo ATCC 27043@ZP_08730919
Vibrio coralliilyticus ATCC BAA 450@ZP_05885804
Marinomonas mediterranea MMB 1@ADZ91901
Burkholderia glumae BGR1@YP_002909241
Ralstonia syzygii R24@CCA86915
Burkholderia thailandensis MSMB43@ZP_02461983
Burkholderia oklahomensis EO147@ZP_02354115
Burkholderia oklahomensis C6786@ZP_02361292
Burkholderia gladioli BSR3@AEA62617
Azospirillum brasilense Sp245@CCD03673
Azospirillum brasilense Sp245@CCD03671
Azospirillum brasilense Sp245@CCD03670
Xanthomonas gardneri ATCC 19865@ZP_08181385
Xanthomonas campestris pv campestris str ATCC 33913@NP_638256
Xanthomonas campestris pv campestris str 8004@YP_242290
Xanthomonas campestris pv vasculorum NCPPB 702@ZP_06486467
Xanthomonas oryzae pv oryzicola BLS256@AEQ97370
Xanthomonas oryzae pv oryzae PXO99A@YP_001914301
Xanthomonas perforans 91 118@ZP_08187121
Xanthomonas campestris pv vesicatoria str 85 10@YP_364948
Xanthomonas fuscans subsp aurantifolii str ICPB 11122@ZP_06705918
Xanthomonas axonopodis pv citri str 306@NP_643396
Ruegeria pomeroyi DSS 3@YP_165226
Ruegeria pomeroyi DSS 3@YP_165225
Rhodobacterales bacterium HTCC2150@ZP_01741096
Pseudovibrio sp JE062@ZP_05083855
Pseudovibrio sp JE062@ZP_05083799
Pseudovibrio sp JE062@ZP_05083514
Pseudovibrio sp JE062@ZP_05083395
Pseudomonas fluorescens Pf 5@YP_257330
Pseudomonas fluorescens Pf 5@YP_257329
Pseudomonas aeruginosa UCBPP PA14@YP_790355
Pseudomonas aeruginosa PACS2@ZP_01366286
Pseudomonas aeruginosa LESB58@YP_002439850
Pseudomonas aeruginosa UCBPP PA14@YP_790354
Pseudomonas aeruginosa PACS2@ZP_01366287
Pseudomonas aeruginosa LESB58@YP_002439849
Pseudomonas aeruginosa 39016@ZP_07794130
Roseibium sp TrichSKD4@ZP_07661112
Roseibium sp TrichSKD4@ZP_07661110
Polymorphum gilvum SL003B 26A1@ADZ69856
Polymorphum gilvum SL003B 26A1@ADZ69857
Labrenzia alexandrii DFL 11@ZP_05116504
Chromobacterium violaceum ATCC 12472@NP_901057
Chromobacterium violaceum ATCC 12472@NP_900394
Chromobacterium violaceum ATCC 12472@NP_900393
Chromobacterium violaceum ATCC 12472@NP_900392
Chromobacterium violaceum ATCC 12472@NP_900391
Burkholderia sp CCGE1003@YP_003910734
Burkholderia sp CCGE1003@YP_003910733
Burkholderia ubonensis Bu@ZP_02381584
Burkholderia sp 383@YP_366670
Burkholderia ambifaria MEX 5@ZP_02905801
Burkholderia ambifaria MC40 6@YP_001807947
Burkholderia ambifaria IOP40 10@ZP_02889070
Burkholderia ambifaria AMMD@YP_773105
Burkholderia sp 383@YP_366668
Burkholderia ambifaria MEX 5@ZP_02905799
Burkholderia ambifaria MC40 6@YP_001807945
Burkholderia ambifaria IOP40 10@ZP_02889068
Burkholderia ubonensis Bu@ZP_02381586
Burkholderia ubonensis Bu@ZP_02381585
Burkholderia sp 383@YP_366669
Burkholderia ambifaria MEX 5@ZP_02905800
Burkholderia ambifaria MC40 6@YP_001807946
Burkholderia ambifaria IOP40 10@ZP_02889069
Burkholderia ambifaria AMMD@YP_773104
Burkholderia ambifaria AMMD@YP_773103
Brevundimonas sp BAL3@ZP_05033395
Brevundimonas sp BAL3@ZP_05033545
Brevundimonas sp BAL3@ZP_05031814
Oceanicaulis alexandrii HTCC2633@ZP_00953290
Kordia algicida OT 1@ZP_02161961
Kordia algicida OT 1@ZP_02161960
Kordia algicida OT 1@ZP_02161959
Azorhizobium caulinodans ORS 571@YP_001526702
Azorhizobium caulinodans ORS 571@YP_001526698
Burkholderia thailandensis MSMB43@ZP_02461981
Rhodospirillum centenum SW@YP_002298200
Rhodospirillum centenum SW@YP_002298201
Rhodospirillum centenum SW@YP_002298202
Rhodospirillum centenum SW@YP_002298203
Rhodospirillum centenum SW@YP_002298204
Rhodospirillum centenum SW@YP_002298205
0.4

Figure S1 Phylogeny of Reb homologues. Unrooted Bayesian phylogenetic tree of 203 Reb homologues. The tree was obtained by using Phylobayes 3.3 with the LG model of amino acid substitution and a discrete gamma distribution with four categories to take into account among-site rate variation. Note that the tree is not fully resolved due to the small number of positions that could be used for analysis (73 amino acids). Numbers at nodes represent posterior probabilities. The scale bar represents the average number of substitutions per site. Colored taxa correspond to the rebs represented in Figure 5.
